# Zenker’s peroral endoscopic myotomy with myomectomy and ablation: a minimally invasive and effective technique for Zenker’s diverticulum resolution

**DOI:** 10.1016/j.vgie.2025.10.009

**Published:** 2025-10-30

**Authors:** Oscar Victor Hernández Mondragón, Kevin Josue Pintor Belmontes

**Affiliations:** Division of Endoscopy, Specialties Hospital, National Medical Center Century XXI, Mexico City, Mexico

## Abstract

**Background and Aims:**

Zenker's peroral endoscopic myotomy (Z-POEM) for Zenker's diverticulum is an effective first-line treatment; however, residual dysphagia may persist because of a remaining mucosal pouch. Currently, no standardized endoscopic method specifically addresses this issue. We present a novel approach: Z-POEM with adjunctive myomectomy and mucosal ablation (Z-POEM-MA), designed to achieve complete anatomical resolution in a single session.

**Methods:**

A 67-year-old man with progressive dysphagia and recurrent aspiration pneumonia was found to have a 4-cm Zenker’s diverticulum. He underwent Z-POEM-MA, which included standard submucosal tunneling and cricopharyngeal myotomy, followed by myomectomy using a polypectomy snare, and subsequent mucosal ablation with argon plasma coagulation.

**Results:**

The procedure lasted 46 minutes with no adverse events. The patient resumed oral intake within 48 hours. Postoperative barium esophagram 1 month postoperatively showed complete resolution of the diverticulum. At 6 months, the patient remained asymptomatic, and upper endoscopy confirmed the absence of recurrence.

**Conclusions:**

Z-POEM-MA is a feasible, safe, and potentially more effective technique compared with conventional Z-POEM, which targets both the muscular and mucosal components of Zenker’s diverticulum. This approach may improve outcomes in patients with large pouches. Further studies are warranted to validate its long-term efficacy and reproducibility.

## Background

**Figure 1:**
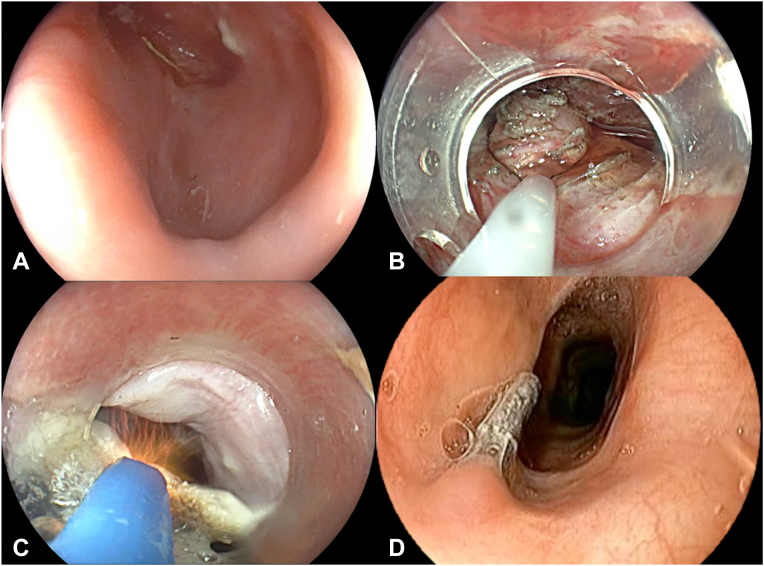
Steps of Zenker's peroral endoscopic myotomy with adjunctive myomectomy and mucosal ablation. **A,** A 4-cm Zenker’s diverticulum sac is observed. **B,** Myomectomy of the septum posterior to a bilateral myotomy. **C,** Ablation of the remnant mucosal pouch. **D,** Absence of Zenker’s diverticulum during follow-up.

Zenker's peroral endoscopic myotomy (Z-POEM) for Zenker's diverticulum is a safe and effective first-line therapy, with high clinical success (>95%), low adverse event rates (5%-8%), and mid- to long-term recurrence of 6% to 8%.^1^^,^^2^ Nevertheless, some patients continue to experience dysphagia, often due to a residual mucosal pouch not addressed by standard techniques.^2^^,^^3^ Recent technical modifications have targeted this component with promising results.^4^ We introduce Zenker's peroral endoscopic myotomy with adjunctive myomectomy and mucosal ablation (Z-POEM-MA), a novel single-session approach addressing both muscular and mucosal components of the diverticulum (Fig. 1).

## Case presentation

**Figure 2:**
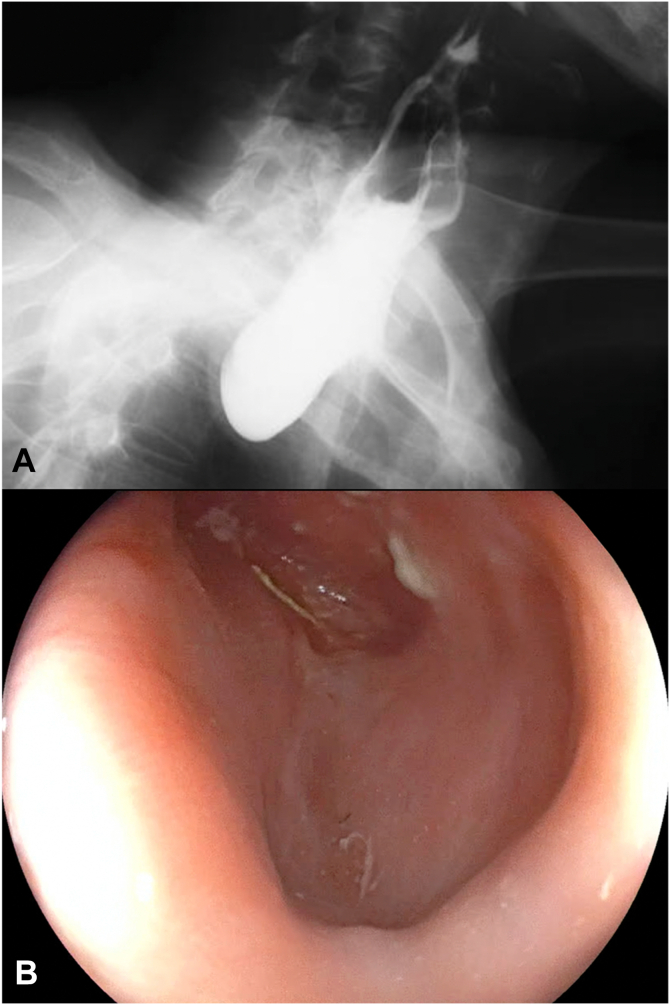
**A,** Barium esophagram showed a 4-cm Zenker’s diverticulum. **B,** Endoscopic view.

**Figure 3:**
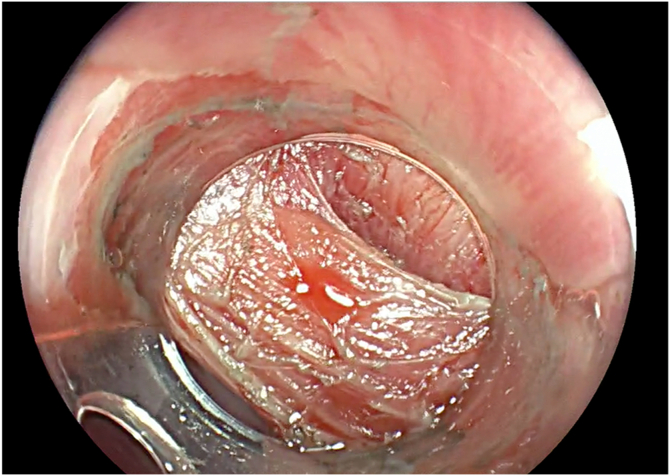
Endoscopic view of cricopharyngeal muscle after completing the bilateral submucosal tunneling.

**Figure 4:**
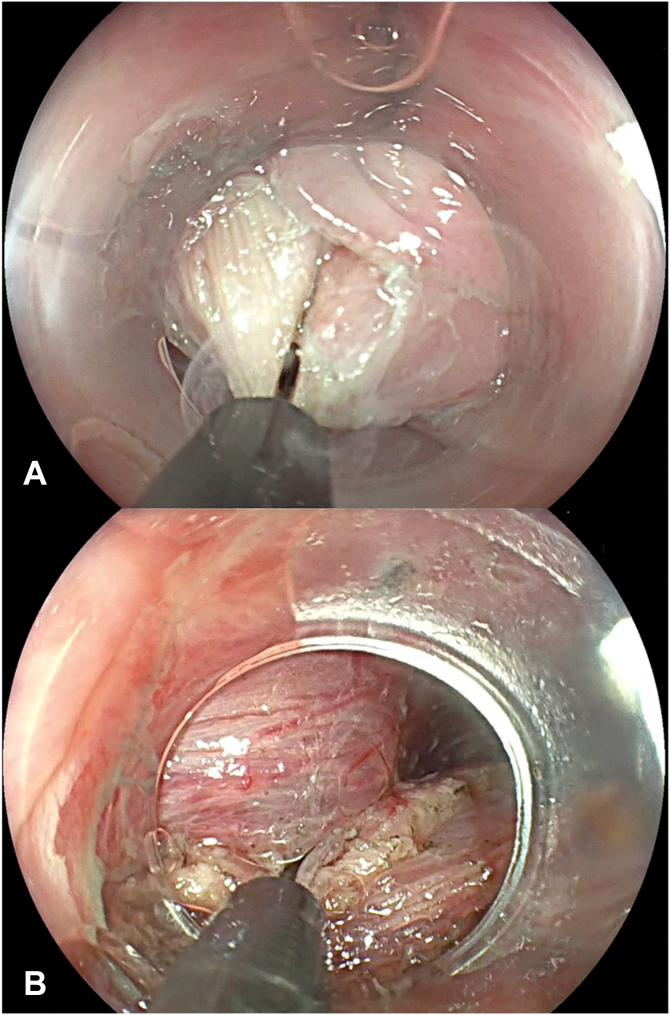
**A,** First myotomy, performed at the right side of the septum. **B,** Second myotomy, performed 1 cm from the previous 1 at the left side.

**Figure 5:**
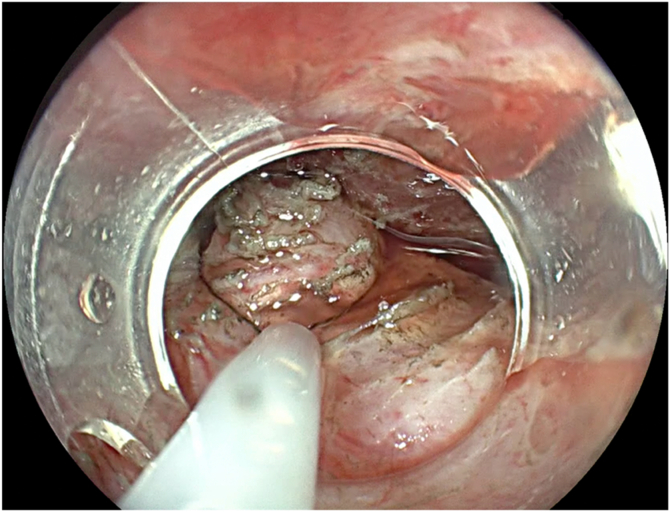
A polypectomy snare was used for the myomectomy of the septum.

**Figure 6:**
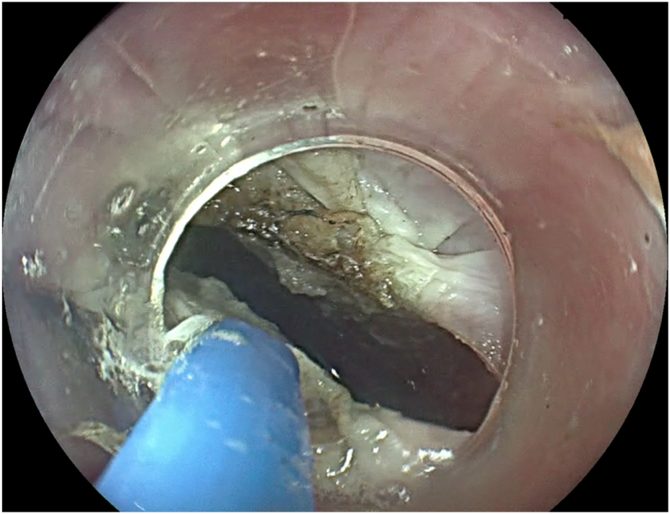
Endoscopic view of the mucosal ablation step using an argon plasma coagulation probe.

A 67-year-old man presented with progressive oropharyngeal dysphagia and chronic cough, and a medical history of multiple hospitalizations due to aspiration pneumonia. A barium esophagram showed a 4-cm Zenker’s diverticulum (Fig. 2). His baseline Dakkak and Bennett dysphagia score was 3.^5^ After discussing risks and benefits of this new endoscopic method, the patient accepted the Z-POEM-MA. This technique was performed with the patient under general anesthesia. A high-definition gastroscope (EG-760; Fujifilm, Tokyo, Japan) fitted with a transparent cap was used. Initially, a conventional Z-POEM technique was performed using an over-the-septum approach. A longitudinal 20-mm mucosal incision was performed using a Triangle Tip Knife (KD-640L; Olympus, Center Valley, Pa, USA) in EndoCut Q mode, effect 3, with a VIO 300 D surgical generator (Erbe, Tübingen, Germany). After bilateral submucosal tunneling and total exposure of the cricopharyngeal muscle (Fig. 3), Z-POEM-MA was performed based on 3 steps: (1) two lateral myotomies, with 1 cm of distance between them (Fig. 4), (2) a myomectomy of the residual muscle with a 13-mm oval stiff polypectomy snare (Captivator II; Boston Scientific, Alajuela, Costa Rica) between the myotomies (Fig. 5), and (3) mucosal ablation up to the diverticular base using argon plasma coagulation (APC) with a FiAPC probe (Erbe) in forced mode, 50 W, effect 4 (Fig. 6). Finally, mucosal closure was performed using 8 hemostatic clips (Resolution 360; Boston Scientific).

**Figure 7:**
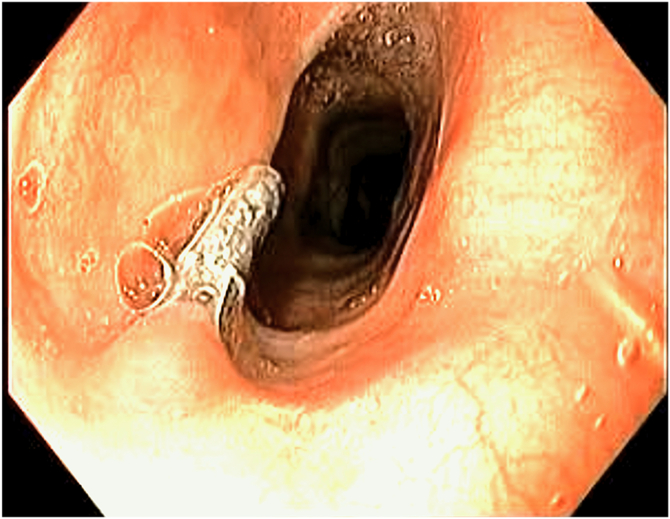
Control upper endoscopy at 1 month after Zenker’s peroral endoscopic myotomy with adjunctive myomectomy and mucosal ablation, showing a complete remodeling and disappearance of the Zenker’s diverticulum and a residual hemostatic clip.

The total procedural time was 46 minutes, with no adverse events during or after the procedure. The barium esophagram at 24 hours showed no leakage, and a liquid diet was initiated and gradually advanced over the following days. The patient was discharged after 48 hours without the need for analgesic medication. At the 1-month follow-up, barium esophagram and upper endoscopy confirmed complete resolution of the Zenker’s diverticulum with no visible pouch (Fig. 7). The patient reported experiencing full symptomatic relief, with a Dakkak and Bennett score of 0. After 6 months, the patient remained asymptomatic with no clinical or radiologic evidence of diverticulum (Video 1, available online at www.videogie.org).

## Conclusions

Z-POEM-MA is a novel, safe, and effective endoscopic technique that addresses both the muscular and mucosal components of Zenker’s diverticulum. Its advantages include single-session performance, the use of only a polypectomy snare and an APC probe, and a modest increase in procedural time (∼15 minutes). Potential drawbacks are the risk of inadvertent mucosotomy or bleeding during myomectomy, possible air-related adverse events with prolonged APC use, and the need for more clips than conventional Z-POEM due to closure of a larger mucosal defect. Further studies with longer follow-up are required to confirm reproducibility, safety, and long-term efficacy. If validated, Z-POEM-MA may offer a more effective option than conventional Z-POEM, particularly in patients with large diverticular pouches.

## Patient Consent

Written informed consent was obtained from the patient for publication of this study.

## Disclosure

All authors disclosed no financial relationships.

## Supplementary data

Video 1 The different steps of the Zenker's peroral endoscopic myotomy with myomectomy and ablation technique.
